# Coverage of education and training of traumatic brain injury-induced growth hormone deficiency in US residency and fellowship programs: a cross-sectional study

**DOI:** 10.1186/s12909-024-05027-8

**Published:** 2024-01-10

**Authors:** Javier Cárdenas, Nicky Kelepouris, Radhika Adiga, Kevin C. J. Yuen

**Affiliations:** 1 Rockefeller Neuroscience Institute, West Virginia University, 33 Medical Center Dr, Morgantown, WV 26506 USA; 2 Novo Nordisk Inc, 800 Scudders Mill Rd, Plainsboro, NJ 08536 USA; 3 Barrow Neurological Institute, 240 W Thomas Rd, Suite 404, Phoenix, AZ 85013 USA

**Keywords:** Traumatic brain injury, Growth hormone deficiency, Fellowship, Residency training, Postgraduate medical education

## Abstract

**Background:**

Hypopituitarism, including growth hormone deficiency (GHD), is a common sequela of traumatic brain injury (TBI). This study explored the coverage of education and training of TBI-induced hypopituitarism in general and GHD in particular, in postgraduate program curricula to identify knowledge gaps and opportunities.

**Methods:**

An online survey and qualitative interviews (focus groups) were conducted among endocrinology, neurology, and physiatry postgraduate program directors in the United States (US). The study received an IRB exemption.

**Results:**

A total of 419 fellowship and residency programs were invited to participate; 60 program directors completed the survey and 11 of these participated in the focus groups. About half of the respondents considered TBI-induced hypopituitarism important or fairly important to include in the curriculum, and nearly two-thirds considered it an appropriate training component. Neurology program directors considered education regarding hypopituitarism following TBI less important and relevant for their curricula compared with endocrinology and physiatry program directors. About half (53%) of the programs responded that they included TBI-induced pituitary disorders in their curricula. About two-thirds (68%) of endocrinology programs, compared with only one-quarter (25%) of neurology programs, covered TBI-induced pituitary disorders. Respondents identified multiple barriers to expanding hypopituitarism following TBI in the curriculum, including the rarity of condition and lack of time/room in the curriculum. Respondents reported that consensus clinical guidelines and the availability of more data on TBI-induced hypopituitarism, including GHD, would greatly impact the development of educational curricula on this topic.

**Conclusions:**

To improve the management of TBI-induced hypopituitarism, education and training should be expanded in US fellowship and residency programs to prepare trainees to effectively screen, diagnose, and treat TBI-induced hypopituitarism, including GHD.

**Supplementary Information:**

The online version contains supplementary material available at 10.1186/s12909-024-05027-8.

## Plain language summary

A traumatic brain injury (TBI) can occur with a sudden blow to the head or the body. Most people recover from TBI within weeks, but the injury can cause long-term effects by reducing the body’s production of growth hormone (GH), which can interfere with daily activities and impair quality of life. This study explored education and training of doctors in the US to identify gaps in knowledge about GH deficiency and opportunities for improvement. Online survey and interviews (focus groups) were conducted among directors of 3 postgraduate (after medical school) training programs: endocrinology, neurology, and physiatry (the diagnosis, prevention, and treatment of all types of impairment related to the brain, nerves, bones, and muscles).

A total of 60 program directors completed the survey and 11 of these participated in the focus groups. About half of the respondents felt education about GH deficiency caused by TBI is important, and nearly two-thirds thought it was appropriate to include in medical training. Half of the programs said that hormone disorders caused by TBI were currently included in their training. Respondents identified multiple barriers to expanding education on this topic in training programs. The main barriers were that the condition is thought to be uncommon and not having time for more training. Respondents thought that clinical guidelines and availability of more information on the condition would greatly impact the development of training about GH deficiency after TBI.

To improve the management of GH deficiency caused by TBI, education and training should be expanded to prepare doctors in training to be better able to screen, diagnose, and treat GH deficiency caused by TBI.

## Background

Traumatic brain injury (TBI), a leading cause of morbidity and mortality [1–3], is often accompanied by systemic conditions through its impact on the pituitary gland. TBI occurs in about 2.9 million children and adults in the United States (US) each year [3]. Symptoms of pituitary gland injury and dysfunction overlap with those of TBI, increasing the difficulty of detecting hypopituitarism [4, 5]. Deficiency of one or more anterior pituitary hormones is present in 30–35% of patients with TBI [4, 6, 7].

Among the anterior pituitary hormonal disorders following TBI, deficiency of growth hormone (GH) has been commonly identified across clinical epidemiological studies [8–12]. Acute growth hormone deficiency (GHD) resulting from TBI-induced hypopituitarism has been reported to occur in 2–30% of patients within one month of TBI [8, 11, 12]. Chronic TBI-induced GHD (defined as at least 6 months after the injury) occurs in 10–63% of patients [12–14]. The wide variance in estimates can be attributed to the timing of the assessment, injury severity, age of onset, and the methods used to diagnose/confirm pituitary hormone dysfunction. Additionally, most of the literature on TBI and TBI-induced GHD describes patients who required hospital admission, typically with moderate or severe TBI. Since most TBIs are mild and do not result in hospital admission, the prevalence of TBI is likely underestimated [15, 16].

Even milder forms of TBI can be accompanied by GHD [17], and its prevalence increases with co-occurrence of other chronic conditions [18]. The prevalence of GHD appears to be associated with trauma severity and has been reported in nearly 15% of patients following repetitive mild head trauma [19]. GHD following TBI seems to confer a worse prognosis than TBI without GHD [20]. In two small studies, GH replacement has been shown to provide long-term metabolic and probable neurocognitive benefits in patients with mild TBI and GHD [21, 22].

Despite broader recognition based on the clinical studies, the wide reported prevalence of GHD suggests under-identification or underdiagnosis. Delayed diagnosis of GHD following TBI results in worse outcomes and hampers rehabilitation and recovery [5, 23]. Specialists treating TBI rarely refer patients to an endocrinologist [5].

Besides improving patient access to specialist care, clinical education and attention to the possibility of GHD among patients with TBI can enhance the likelihood of its timely identification and treatment. Therefore, this study aims to understand the extent to which educational exposure to TBI-induced hypopituitarism, including GHD, is provided in the training of specialist physicians. We sought to (1) understand and quantify how hypopituitarism/GHD-specific training is currently offered in residency/fellowship programs, (2) identify gaps between key competencies and current residency and fellowship programs, and (3) uncover barriers and opportunities to improve the inclusion of leading practices in the early identification and treatment of hypopituitarism and GHD in patients with TBI in training programs.

## Methods

### Study design and participants

Between January 19, 2022, and March 28, 2022, an online survey was conducted among endocrinology, neurology, and physiatry residency and fellowship program leaders. A list of 419 US residency or fellowship programs was compiled from the American Medical Association’s FREIDA™ database [24], of which 167 were endocrinology, 171 were neurology, and 81 were physiatry programs. Program leaders were identified for all of the residency and fellowship programs through the FREIDA™ database and further online searches of the individual programs. Endocrinology included both adult and pediatric programs.

Using the compiled list of 419 programs, participants were recruited via email and postal invitations, which included information on study objectives, study sponsor, and a link to the anonymous online survey. Participants were offered a modest honorarium of $125 for completing the survey. Respondents who selected yes (from yes/no options) indicating consent to participate in the study were allowed to enter the screening portion of the survey. Participants with adequate knowledge about their residency or fellowship program curriculum were asked to complete the survey. Email reminders to non-responders were sent twice after the first mailing. Responses were restricted to one per institution to ensure data consistency and representation across institutions. The WCG Institutional Review Board reviewed the study and qualified it for exempt status.

The survey (Additional file 1), developed by KJT Group under the direction of the authors, included approximately 36 yes/no, single-select, multiple-choice, and Likert-scale questions. Survey questions included the structure of the program, description of how GHD and TBI are addressed in the curriculum, and an assessment of barriers to, and opportunities for, including these topics in the program. Four-point Likert scales were used to assess importance, appropriateness, responsibility, preparedness, impact (very, fairly, somewhat, not at all important/appropriate/responsible/prepared/impactful), extent (great extent, some extent, very little extent, and not at all), influence (great deal, some, very little, no influence at all), and barriers (not a barrier, small, moderate, large barrier).

Following the initial quantitative survey, 90-minute qualitative interviews in the form of two online focus groups were conducted between September 7 and September 13, 2022, with some of the respondents who completed the online survey. At the end of the survey, respondents were asked about their willingness to participate in focus groups in the next few months to learn more about the state of education related to endocrine and neurological conditions in residencies/fellowships in the United States; those interested were invited to participate in the focus groups. The purpose of the qualitative portion of the study was to validate the survey findings and obtain additional insights.

### Statistical analyses

For the quantitative data, descriptive statistical analysis (means, frequencies) of the aggregated data was performed using Q Research Software for Windows 23 (A Division of Displayr, Inc., New South Wales, Australia). Except as noted otherwise, categorical data as percentages and continuous data as mean values are presented. The qualitative data was analyzed thematically. The analysis consisted of coding the transcripts to identify emerging themes and constructs.

## Results

### Sample characteristics

Of the 419 programs contacted, 60 respondents completed the online survey: 28 in endocrinology, 24 in neurology, and eight in physiatry programs, yielding a response rate of 14%. The median survey length was 12 min. Program specialties included endocrinology (36%), all of which were fellowship programs; neurology (45%), all of which were residency programs; and physiatry (18%), which primarily consisted of residency programs. All programs were adult practice. The qualitative phase of this research included 11 programs that completed the 90-minute interview. The first group included one endocrinology program and three neurology programs. The second group included three endocrinology programs, two neurology programs, and two physiatry programs.

Most survey respondents were program directors (87%); 8% were associate or co-program directors and 5% were assistant directors. Respondents most frequently reported that teaching occupied some of their time (60%) or most of their time (38%). See Table 1 for sample and program characteristics. Most programs were university-based or affiliated with a university. Table 2 presents a comparison of characteristics by program type, compared with all 419 US programs.

**Table 1:** Sample characteristics

Characteristics of Survey Respondents	Total Respondents (*N* = 60)
**Region**, ***n*(%)**	
Northeast	22 (37)
South/Mid-Atlantic	17 (28)
North Central	11 (18)
West	10 (17)
**Program specialty**, ***n*(%)**	
Endocrinology	28 (47)
Neurology	24 (40)
Physiatry	8 (13)
**Program type**	
Endocrinology	100% fellowship
Neurology	100% residency
Physiatry	88% residency, 13% fellowship
**Practice type**, ***n*(%)**	
Adult practice	60 (100)
**Role**, ***n*(%)**	
Program director	52 (87)
Associate or co-program director	5 (8)
Assistant director	3 (5)
Chief resident	0 (0)
**Mean time at institution, years**	13.2 (median 13, range 1‒44)
**Mean time in current role, years**	7.1 (median 6, range 1‒28)
**Professional time spent teaching**, ***n*(%)**	
Almost all of my time	0 (0)
Most of my time	23 (38)
Some of my time	36 (60)
Very little of my time	1 (2)
None of my time	0 (0)
**Length of program**, ***n*(%)**	
1 year	2 (3)
2 years	26 (43)
3 years	10 (17)
4 years	22 (37)
**Institution type**, ***n*(%)**	
Medical school based	46 (77)
Community, medical school affiliated	6 (10)
Community, nonaffiliated	5 (8)
Community, medical school administered	3 (5)
**Mean number of residents/fellows**	13.1 (median 9, range 1‒48)
**Populations served**, *n*(%)*	
Inner-city	29 (48)
Urban (non-inner city)	30 (50)
Suburban	34 (57)
Rural	13 (22)

*Responses sum to > 100% as respondents could select multiple responses

**Table 2:** Comparison of sample characteristics with US programs, by program type

Residency/Fellowship Programs^		
**Endocrinology**	**Survey Sample** ***n***(***n* = 28**)	**US Endocrinology Programs** **(%)** (***n* = 167**)
Institution type		
Community based	0 (0)	5
Community based; university affiliated	5 (18)	16
Military	0 (0)	1
University based	23 (82)	78
Other	0 (0)	1
**Region**		
Midwest	2 (7)	22
Northeast	13 (46)	30
South	8 (29)	34
West	5 (18)	14
**Neurology**	**Survey Sample** ***n*(%)** (***n* = 24**)	**US Neurology Programs** **(%)** (***n* = 171**)
**Institutiontype**		
Community based	2 (8)	8
Community based; university affiliated	3 (13)	25
Military	0 (0)	1
University based	19 (79)	65
**Region**		
Midwest	9 (38)	25
Northeast	7 (29)	27
South	6 (25)	32
West	2 (8)	15
Physiatry*	**Survey Sample** ***n*(%)** (***n* = 8**)	**US Physiatry Programs** **(%)** (***n* = 83**)
**Region**		
Midwest	1 (13)	29
Northeast	2 (25)	29
South	2 (25)	30
West	3 (38)	12

*There was insufficient information of program type for US physiatry programs. ^Data is from the American Medical Association’s FREIDA™ database [24].

*Note:* Responses may not sum to 100% due to multiple responses allowed or rounding

### Curricula of programs

Over half (53%) of the programs surveyed included TBI-induced pituitary disorders in their curricula. More than two-thirds (68%) of endocrinology programs, compared with only one-quarter (25%) of neurology programs, covered TBI-induced pituitary disorders, usually during teaching in inpatient rotations, dedicated seminars, lectures or conferences, or precepting in outpatient rotations and continuity clinics.

Among endocrinology programs, over half (57%) reported covering TBI-induced GHD in their curricula; however, coverage was rare among neurology programs (8%). TBI-induced GHD was most often taught through dedicated seminars or inpatient rotations.

### Importance and appropriateness of hypopituitarism following TBI in curriculum

**Fig. 1:**
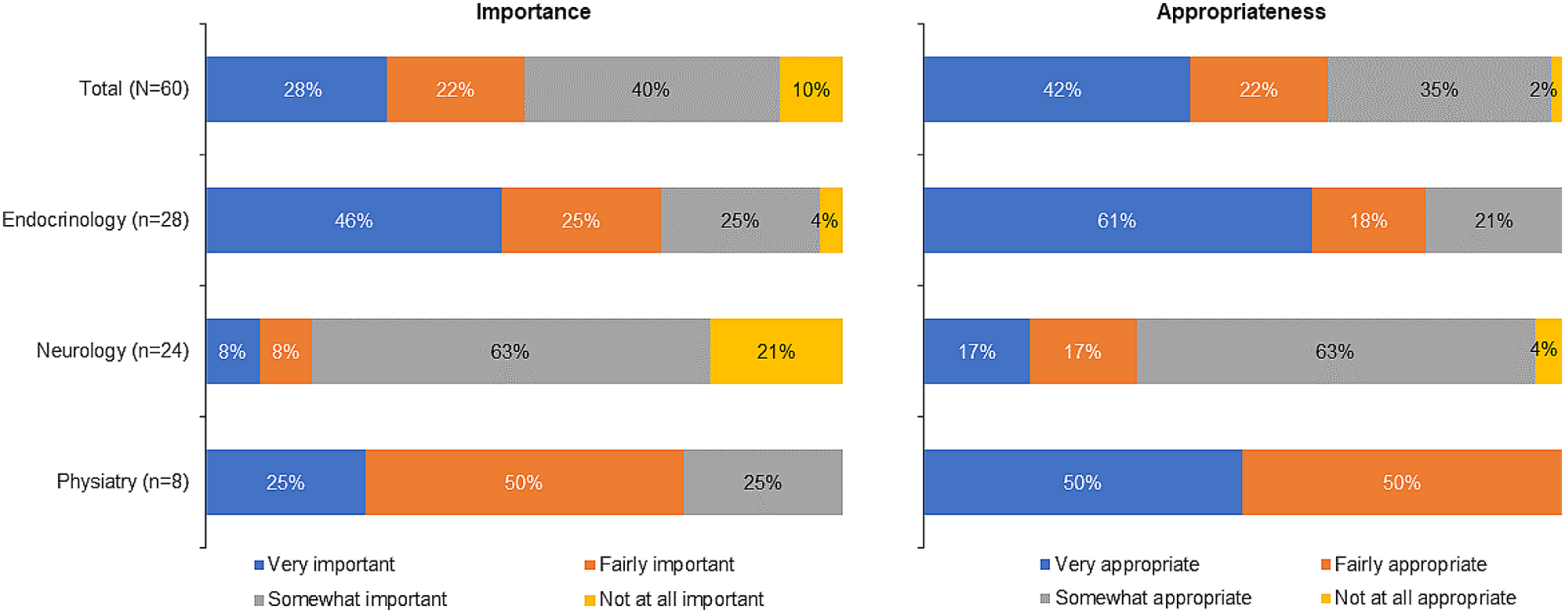
Perceived importance and appropriateness of including hypopituitarism following TBI in the curricula

About half of the respondents thought hypopituitarism following TBI to be important or fairly important to include in the curriculum, and nearly two-thirds considered it an appropriate training component. Neurology programs considered education regarding hypopituitarism following TBI far less important and appropriate for their curricula than endocrinology and physiatry programs (Fig. 1). In the qualitative focus groups, all specialties agreed with the importance of delivering education on TBI-induced GHD, particularly the understanding that TBI can lead to GHD. Neurology participants considered education regarding hypopituitarism following TBI to be far less important and appropriate for their curricula compared with endocrinology and physiatry programs.

### Perceived responsibility of TBI-induced GHD management

**Fig. 2:**
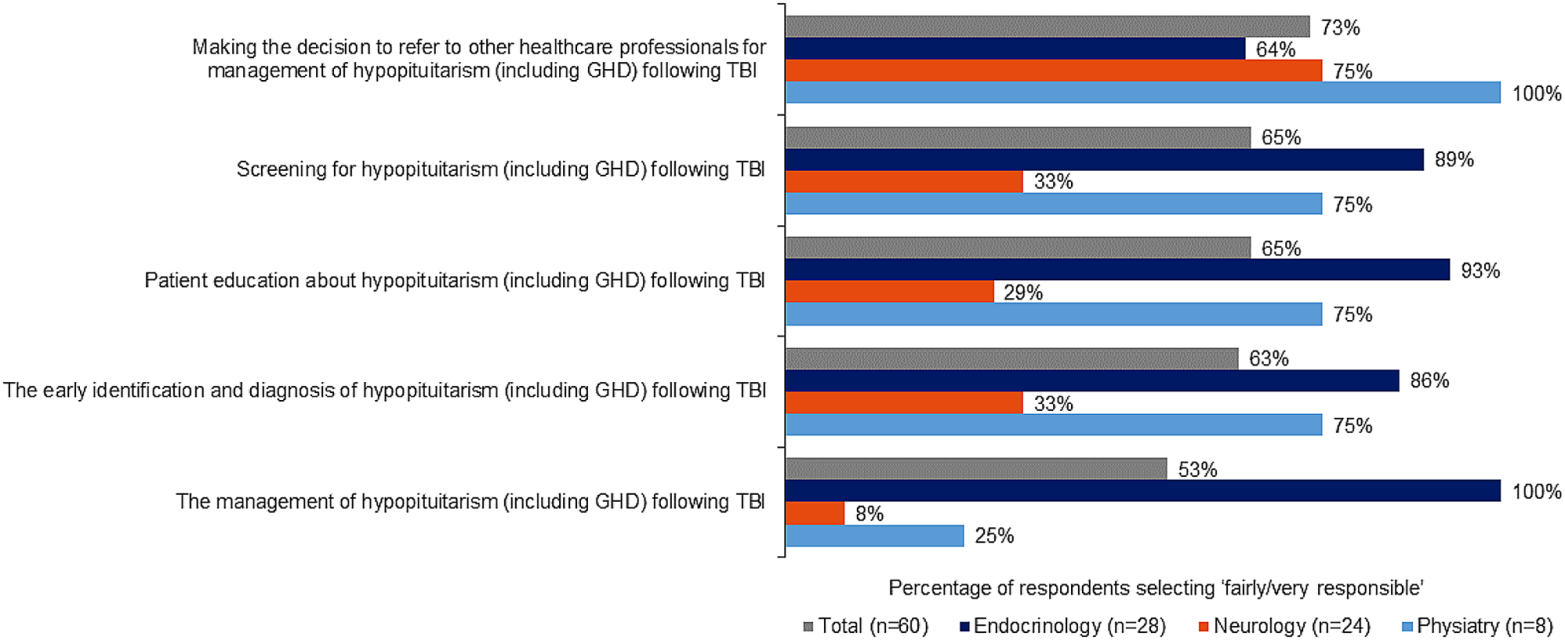
Level of specialist responsibility regarding hypopituitarism following TBI

Endocrinology directors strongly believed endocrinologists are responsible for diagnosing and managing TBI-induced hypopituitarism (including GHD), whereas neurology directors believed neurologists primarily should be responsible for referring appropriate patients. Additionally, endocrinologists felt the greatest responsibility for screening and patient education (Fig. 2). This was confirmed in the qualitative research. Endocrinology was considered as the ‘owner’ of patient diagnosis and management of TBI-induced hypopituitarism. Training in neurology and physiatry programs was reported to be generally limited to the symptoms and making appropriate referrals to endocrinology for follow-up care of TBI-induced GHD. As one neurologist stated, “Things like GH deficiency, not quite on my radar… if I suspect it, I’m probably just going to refer them to an endocrinologist. Most general neurologists, are probably not thinking about GHD, unless they have that subspecialty type training or that familiarity.”

## Extent of coverage of GHD topics in program curricula

**Fig. 3:**
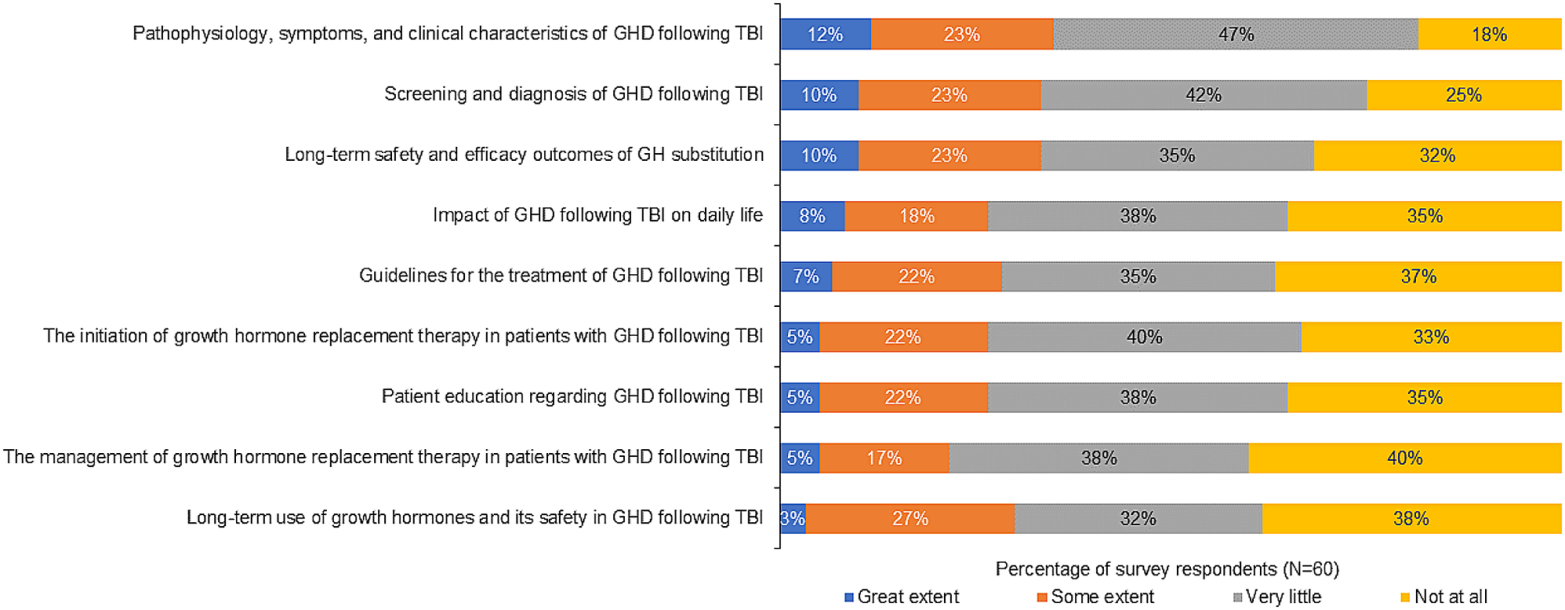
Coverage of GHD topics across all programs (endocrinology, neurology, and physiatry)

The majority of respondents reported that the listed GHD topics received very little or no coverage in the curriculum. Topics covered to the greatest extent among residency/fellowship program curricula were TBI-induced GHD pathophysiology (35% some/great extent), screening and diagnosis (33%), and long-term safety and efficacy outcomes of GH substitution (33%)(Fig. 3).

According to participants in the focus groups, hypopituitarism education includes GHD as a subset of training for endocrinology programs, but to a lesser extent than TBI for neurology and physiatry programs. GHD and TBI are addressed with didactic lessons as well as clinical rotations. TBI-induced GHD is not a subject of specific training as residents/fellows are expected to garner this knowledge through a small portion of the larger lessons on TBI or GHD, or exposure through bedside training. Most endocrinology programs included GHD competencies to a small extent, particularly competencies regarding the long-term use of growth hormones, screening and diagnosis, and GHD pathophysiology. Patient education regarding GHD following TBI and the management of GH replacement therapy in patients with GHD following TBI received the least coverage (Supplementary Fig. 1 in Additional file 2). Among neurology programs, curriculum coverage of TBI-induced GHD is sparse; most programs have no time allotted for diagnosis, management, and treatment of GHD (Supplementary Fig. 2 in Additional file 2).

### Preparedness to manage TBI-induced GHD

**Fig. 4:**
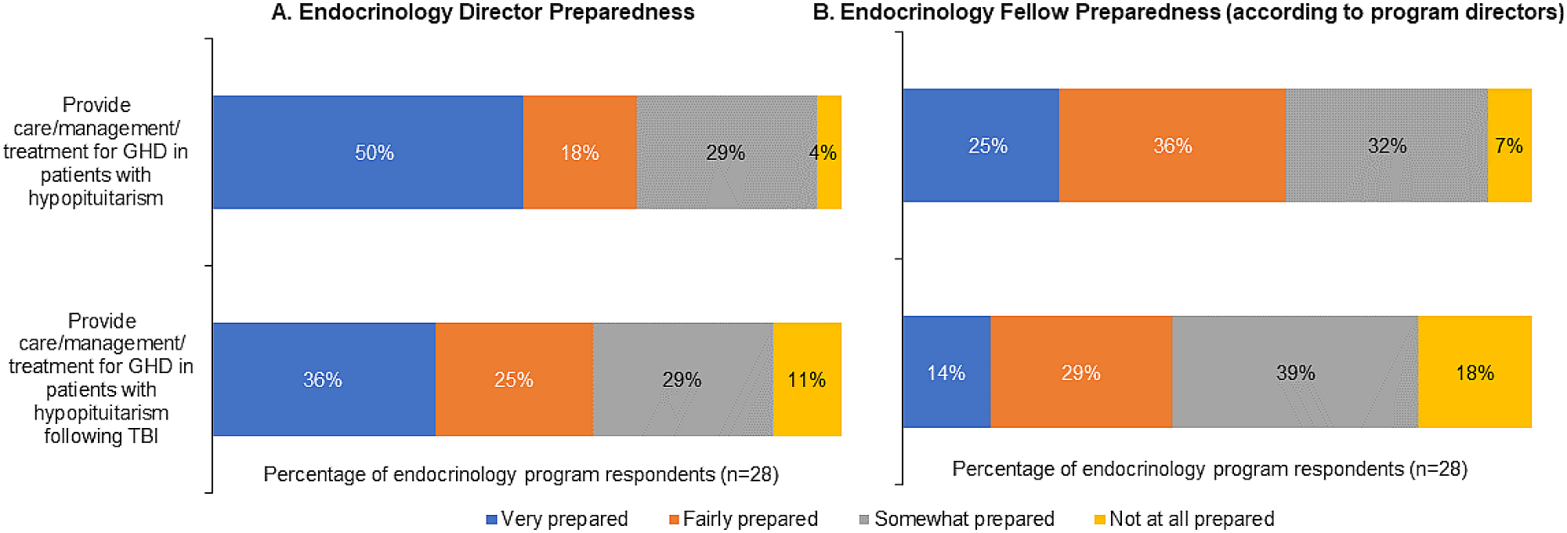
Endocrinology director preparedness (A) and endocrinology fellow preparedness (B) (according to program directors)

**Fig. 5:**
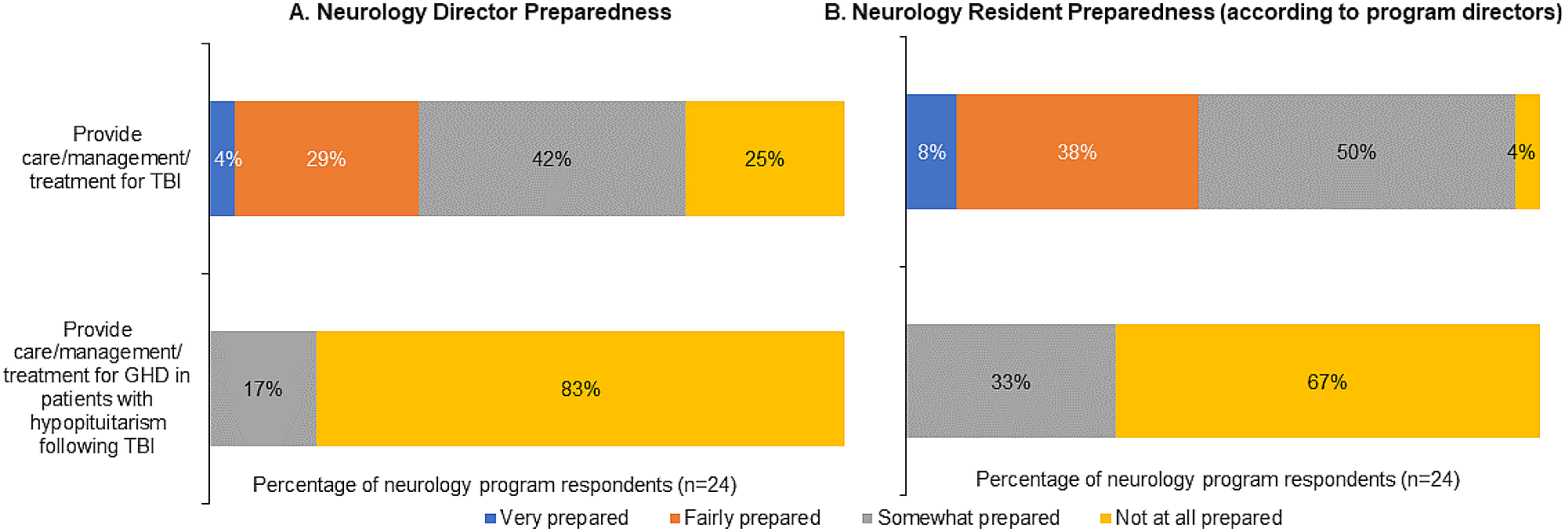
Neurology director preparedness (A) and neurology resident preparedness (B) (according to program directors)

Regarding the level of preparedness to manage GHD and GHD secondary to TBI, endocrinology directors considered themselves relatively more prepared than their graduating fellows. Just over a third reported feeling prepared to provide management for GHD following TBI. Although more than 60% of directors considered fellows to be at least fairly prepared to manage GHD in patients with hypopituitarism, less than half thought fellows were similarly prepared to manage GHD following TBI (Fig. 4). No neurology program leaders felt they or their residents were very or fairly prepared to manage TBI-induced GHD (Fig. 5).

### Plans for expansion of education/training on TBI-induced GHD in curricula and barriers to expansion

**Fig. 6:**
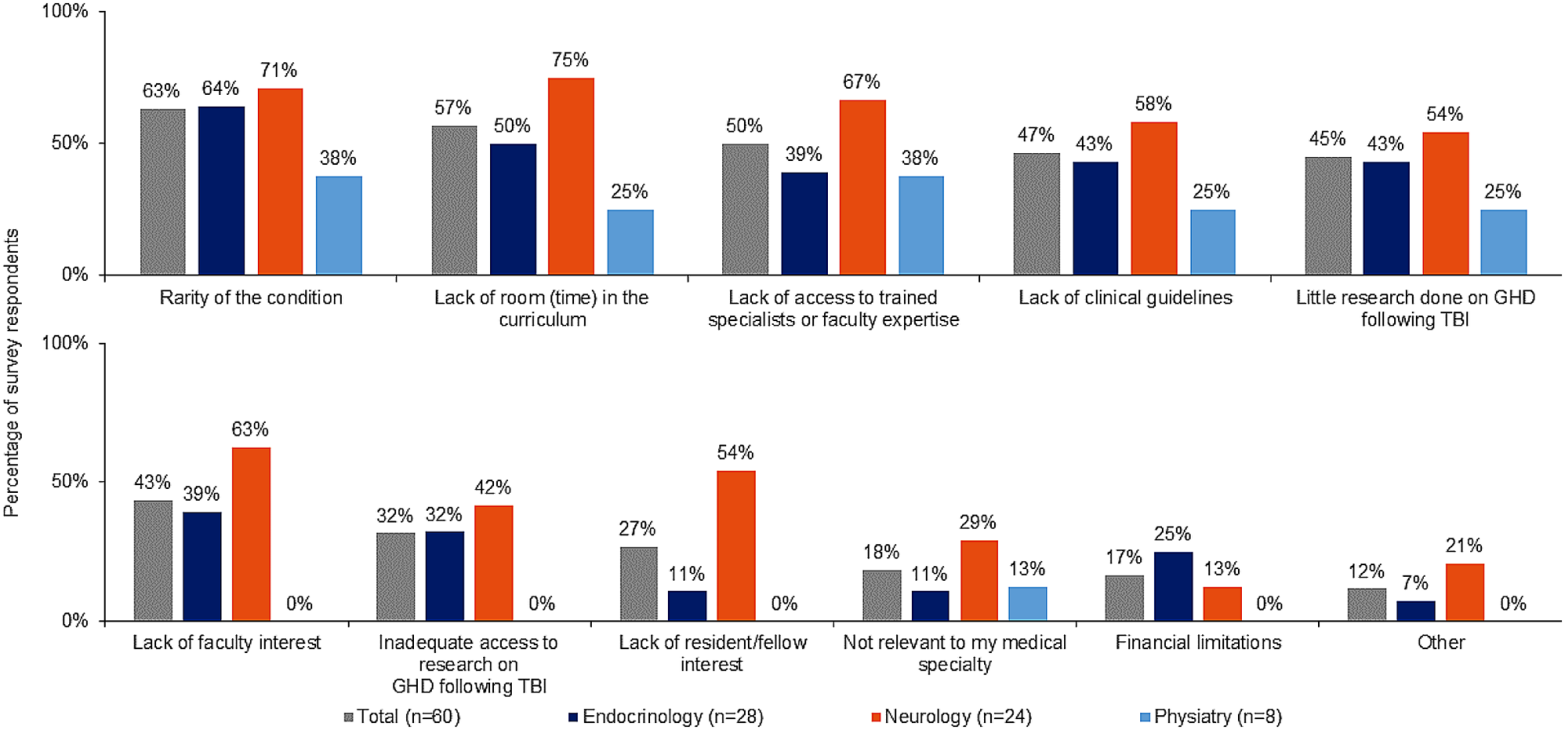
Barriers to integrating hypopituitarism following TBI education

Few programs (22%) planned to expand education or training surrounding TBI-induced hypopituitarism, but this varied by program type: endocrinology, 25%; neurology, 13%; and physiatry, 38%. Of the 13 programs that reported planned expansion, almost half (46%) indicated the curriculum changes were currently in progress at the time the survey was conducted. Respondents identified multiple barriers to expanding hypopituitarism following TBI in the curriculum. Perceived rarity of the condition and lack of room in the curriculum were considered to be the greatest barriers to furthering TBI-hypopituitarism education. Neurology directors perceived lack of room, lack of access to expertise, and lack of interest among faculty/residents as larger barriers relative to directors of endocrinology and physiatry (Fig. 6).

Although most program directors participating in the qualitative research suspected TBI-induced GHD is underdiagnosed, several factors limited the extent of TBI-induced GHD coverage in the curricula: low (perceived) prevalence, vague symptoms and low awareness among neurology and physical medicine and rehabilitation, difficulty in accessing and performing GHD testing, lower morbidity of GHD compared with other hypopituitary conditions, and lack of demand for education among residents/fellows and professors.

### Impact of resources and additional learning opportunities

**Fig. 7:**
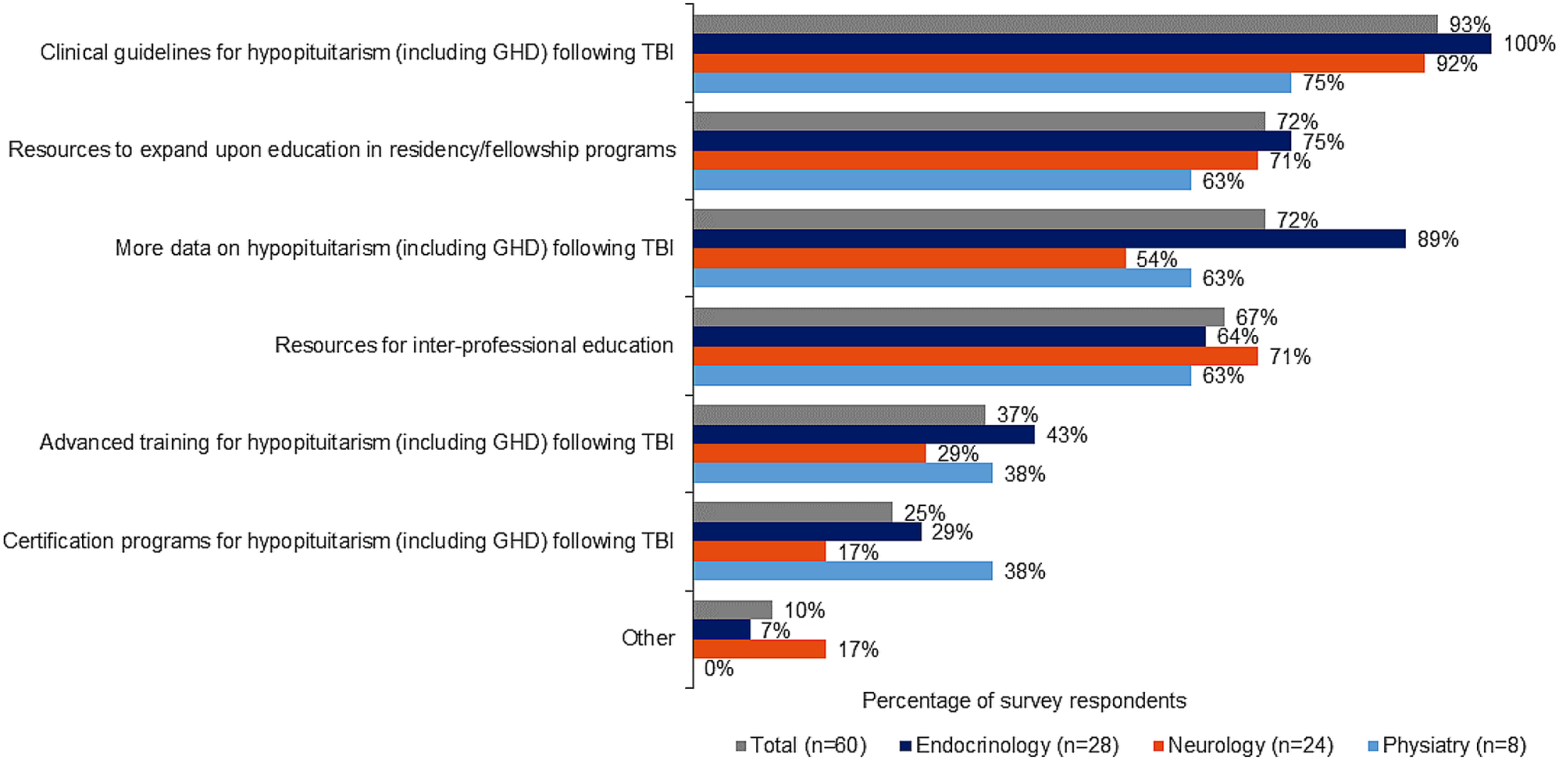
Impact of resources on expanding education

Clinical guidelines topped the list of potential resources having the greatest impact on further TBI-hypopituitarism education. Directors also cited the need for additional research exposure on hypopituitarism, including GHD post TBI, as well as more resources to expand education in residency and fellowship programs (Fig. 7). More than half of respondents said resources for inter-professional education are needed to expand curricula with TBI-hypopituitarism education (Fig. 7). Respondents cited online resources, continuing medical education (CME) courses, and webinars as the most effective learning opportunities; endocrinology directors placed higher value on conferences and professional associations relative to directors of the other two program types (supplementary Fig. 3 in Additional file 2).

The qualitative focus groups revealed additional insights into ways to increase awareness of TBI-induced GHD such as conducting presentations at key conferences and publishing data on outcomes of GHD in patients with TBI across target specialties. Presentations at conferences and published literature providing data on outcomes and prevalence of TBI-induced GHD are key areas of opportunity for raising awareness of the condition. Additionally, developing online content (e.g., webinars, podcasts, CMEs) can offer supplemental education opportunities.

## Discussion

Hypopituitarism, including GHD, is an under-recognized sequela of TBI. More than one-third of patients with TBI develop deficiency of one or more anterior pituitary hormones, with GHD being the most frequent [6, 7, 25]. Our study aimed to explore the education and training opportunities of TBI-induced hypopituitarism, including GHD, in US-based postgraduate training program curricula to identify knowledge gaps and opportunities in this area of patient care. Additionally, identification of barriers to, and opportunities for, improving early identification and application of evidence-based hypopituitarism and GHD management associated with TBI were explored.

We found that of the residency and fellowship programs surveyed, endocrinology program leaders were most likely to believe that endocrinologists were responsible for patient diagnosis and management of TBI-induced hypopituitarism, including GHD. Yet a third of endocrinology programs surveyed do not cover TBI-induced pituitary disorders in their curricula, and 43% do not provide education about GHD following TBI. According to the American Board of Internal Medicine’s Endocrinology, Diabetes, & Metabolism Certification Exam, the topic of hypopituitarism and its etiologies including TBI only constitutes less than 2% of the exam [26]. By contrast, Brain Injury Medicine fellowship milestones from the Accreditation Council for Graduate Medical Education (ACGME) include a requirement to be able to “describe diagnostic and therapeutic measures for secondary complications” as part of “Medical Knowledge 1: Traumatic and Non-Traumatic Brain Injury”, [27] and the American Board of Psychiatry and Neurology Certification Examination in Neurology includes coverage of GH and the pituitary gland as part of neuroendocrinology and a subset of “neuroscience and mechanism of disease.” [28] However, coverage of TBI-induced pituitary disorders in general and GHD following TBI specifically is remarkably lower in neurology programs according to the neurology directors surveyed. More than 70% of endocrinology program directors considered hypopituitarism following TBI to be very or fairly important and appropriate to include in the curricula. In contrast, most neurology directors considered the subject to be only somewhat important or not at all important. Additionally, given that neurologists are often called for in caring for patients with TBI, their awareness and education regarding GHD among patients with TBI is crucial for its early identification and treatment.

This research identifies the need for heightened awareness of TBI-induced hypopituitarism, including GHD. Few fellowships for brain injury management exist, and the number of applicants has trended down [29]. Only 61% of endocrinology directors in our study felt fairly or very prepared to treat GHD in patients with hypopituitarism following TBI, which may impact training. Presentations at key medical conferences and publication of outcomes data on TBI-induced GHD may increase awareness, vigilance, and recognition of this under-recognized condition. Online content (e.g., webinars, podcasts, CMEs) could additionally provide accessible supplemental education opportunities.

### Limitations

The survey used in this research was not validated or pilot tested. The positions and program knowledge of participants are self-reported. There is potential for responder, recall, and selection biases that may limit the generalizability of the findings to the larger population of neurology, endocrinology, and physiatry residency and fellowship programs. Larger programs with multiple directors had a greater opportunity for participation, as we sent survey invitations to all available contacts. A small portion of the fellowship and residency programs responded, and although participation was limited to one representative per institution to ensure as many programs were represented as possible, the survey findings may not be generalizable to other programs, particularly physiatry, because of the very small sample size for this program type in particular. However, despite the low response rate, the geography and program type of the sample is representative of the overall US endocrinology and neurology residency and fellowship programs. The topic of the study, TBI-induced hypopituitarism, may have had a role in respondents’ decision to participate in the survey; those more interested in this condition may have been more likely to participate and thus be disproportionately represented.

We are not aware of any other research that has extensively studied postgraduate medical training programs for curriculum inclusion of hypopituitarism, including GHD, related to TBI. A wide range of research could follow our study to understand greater nuance in current curricula designs. Due to the small sample size, differences between academic and tertiary centers were not assessed. The online focus group, which was quite small with the intention of providing a sample of perceptions, could be expanded to gain greater insight. The current content included in curricula could be examined in detail.

## Conclusions

This research identifies the need for more education on the diagnosis and treatment of TBI-induced hypopituitarism (including GHD) in US endocrinology and neurology residency and fellowship programs, with more research needed to explore training and educational gaps in physiatry programs. Physicians need to be trained to effectively screen, diagnose, and optimally treat their patients with TBI-induced GHD. Greater awareness of TBI-induced GHD may lead to increased coverage of this condition in residency/fellowship curricula. Perceived lack of importance could be the major reason for underdiagnosis. Supplemental education opportunities including online content such as webinars, CMEs, and podcasts, can help address these training needs. Further studies are needed to assess whether providing such supplemental education opportunities will increase awareness and subsequently generate more diagnostic work-ups and endocrinology referrals for hypopituitarism in patients with TBI.

### Electronic supplementary material

Below is the link to the electronic supplementary material.

**Supplementary Material 1: Appendix 1.** TBI and GHD residency curriculum questionnaire

**Supplementary Material 2: Supplementary Figure 1.** Coverage of GHD topics in endocrinology fellowship programs. **Supplementary Figure 2.** Coverage of GHD topics in neurology residency programs. **Supplementary Figure 3.** Most effective additional learning opportunities (outside the standard curriculum). **Graphical Abstract**

## Data Availability

The datasets generated during the current study are not publicly available due to containing proprietary information but are available from the corresponding author on reasonable request.

## List of Abbreviations

CME: continuing medical education
GH: growth hormone
GHD: growth hormone deficiency
TBI: traumatic brain injury
US: United States
