# Supplementary material for: Coverage of education and training of traumatic brain injury-induced growth hormone deficiency in US residency and fellowship programs: a cross-sectional study
Source: BMC Med Educ. 2024 Jan 10;24:53. doi: 10.1186/s12909-024-05027-8 (PMC10782717; doi:10.1186/s12909-024-05027-8)
Supplement: Supplementary file 1 — Supplementary Material 1: Appendix 1. TBI and GHD residency curriculum questionnaire [file 12909_2024_5027_MOESM1_ESM.pdf]

Additional file 1

Appendix 1. TBI and GHD Residency Curriculum Questionnaire

SAMPLE PRELOAD AND SCREENING QUESTIONS

[PRELOAD RESIDENCY/FELLOWSHIP PROGRAMS NAMES AND STATE INFORMATION]

ALL RESPONDENTS
P1 Program ID from contact list

[LIMIT OF 1 RESPONDENT PER SCHOOL]

1. [SCHOOLS FROM CONTACT LIST. POPULATE USING COLUMN D OF EACH TAB – ENDOCRINOLOGY, NEUROLOGY, PHYSIATRY]

[PROGRAMMER NOTE]
IF A RESPONDENT FROM THE SAME INSTITUTION HAS ALREADY COMPLETED THE SURVEY, DISPLAY “Thank you for your interest in this survey. Unfortunately, someone from your institution has already participated in this survey and we are currently limiting responses to one per institution. You may receive survey invitations from us for future studies on relevant topics.”

ALL RESPONDENTS
P2 State from contact list

[COLUMN H IN CONTACT LIST]

ALL RESPONDENTS
P3 Residency/Fellowship program type

[COLUMN V IN CONTACT LIST]
1. Endocrinology
2. Neurology
3. Physiatry

ALL RESPONDENTS
S0 Please provide your contact information below. Your information will only be used by KJT Group for the sole purpose of this survey and will not be shared or used for any marketing activities or for any other purpose. Additionally, your identity will not be associated with your survey responses in any way.

First Name: [MANDATORY TEXT BOX]
Last Name: [MANDATORY TEXT BOX]
Email address: [MANDATORY TEXT BOX]

99. I do not wish to continue; I would like to end my participation here. [TERMINATE]

PROVIDES CONTACT INFORMATION (S0 COMPLETED)

S1 Thank you for your interest in this survey. We appreciate your willingness to participate in this important research on residency and fellowship curricula.

Before participating, KJT Group requires you to review the following information:

- Academic Researchers from Barrow Neurological Institute in collaboration with Novo Nordisk Inc. are conducting this survey to **understand the state of education related to endocrine and neurological conditions in residencies/fellowships in the United States.**
- KJT Group is a **global market research company headquartered in Rochester, NY**.
- You have been invited to participate as you may be familiar with the residency/fellowship curriculum.
- Your participation involves completing this survey.
- This survey will take approximately 15 minutes to complete.
- Your **participation is voluntary,** and you may choose to stop participating at any time (withdraw consent).
- Your responses are **strictly confidential** and no personally identifiable information will be collected.
- Findings of this research may be published in scientific journals or presented at medical meetings. Only aggregated results will be presented; data will never be presented in a way that identifies an individual or the institutions.
- Please be advised that some institutions/universities may restrict engagement in this type of market research. Please refer to your own organization's policies to determine whether it is appropriate for you to participate and receive compensation. In addition, all compensation provided to you based on your participation may be reportable under state and/or federal transparency laws and regulations.
- This survey is determined exempt by the WCG IRB. If you need additional information on this survey or have any questions, please contact help@kjtgroup.com and reference "J21179."
- Note: If you believe that your patient has experienced an adverse event while using Novo Nordisk products, please call (800) 727-6500 to report this event.

If you qualify for and complete this research, you will be eligible to receive the honorarium referenced in your invitation.

Do you consent to these terms and wish to continue?

1. Yes
2. No

[IF CONSENTS (S1r1) CONTINUE, ELSE TERMINATE]

AGREES TO PARTICIPATE (S1r1)
S2 What is your role in the residency/fellowship program?

1. Program Director
2. Associate or co-program director
3. Assistant director
4. Chief Resident
5. Other

[IF DIRECTOR OR CHIEF RESIDENT (S2r1-4) CONTINUE, ELSE MARK UNQUALIFIED AND CONTINUE]

AGREES TO PARTICIPATE (S1r1)
S2B Which of the following best describes the program you are primarily responsible for?

1. Residency program
2. Fellowship program

AGREES TO PARTICIPATE (S1r1)
S3 Which of the following best describes the specialty of your residency/fellowship program?

[ALPHA SORT]
1. Endocrinology
2. Neurology
3. Physiatry/Brain Injury Medicine
4. Other

[IF PROGRAM IS ENDOCRINOLOGY, NEUROLOGY, OR PHYSIATRY, CONTINUE (S3r1-3) CONTINUE, ELSE TERMINATE]

ENDOCRINOLOGY, NEUROLOGY, OR PHYSIATRY RESIDENCY (S3r1-3)
S3B Does your residency/fellowship program focus primarily on adult practice or pediatrics?

1. Adult practice
2. Pediatrics

ENDOCRINOLOGY, NEUROLOGY, OR PHYSIATRY RESIDENCY (S3r1-3)
S4 Are you familiar with the learning objectives and curriculum of your residency/fellowship program?

1. Yes
2. No

[IF FAMILIAR (S4r1) CONTINUE, ELSE TERMINATE]

ALL RESPONDENTS
S100 FINAL QUOTA QUESTION

1. Endocrinology Program Directors [N=50]
    - Agrees to participate (S1r1)
    - Appropriate role/title (S2r1-4)
    - Endocrinology program (S3r1)
    - Familiar with curriculum (S4r1)

2. Neurology Program Directors [N=50]
    - Agrees to participate (S1r1)
    - Appropriate role/title (S2r1-4)
    - Neurology program (S3r2)
    - Familiar with curriculum (S4r1)

3. Physiatry Program Directors [N=25]
    - Agrees to participate (S1r1)
    - Appropriate role/title (S2r1-4)
    - Physiatry program (S3r3)
    - Familiar with curriculum (S4r1)

4. Not qualified [n=9999]

RESIDENCY/FELLOWSHIP BACKGROUND

ALL QUALIFIED RESPONDENTS
Q200 You have qualified for the full survey! Thank you for your responses thus far. The remainder of this survey should take approximately 15 minutes to fully complete.

Please click the forward arrow to continue.

ALL QUALIFIED RESPONDENTS
Q201 How long have you been at your current institution?

[RANGE: 0-50]
[_|_] years

ALL QUALIFIED RESPONDENTS
Q202 How long have you been in your current role as it is related to the [INSERT S3] residency/fellowship curriculum?

[RANGE: 0-Q201]
[_|_] years

ALL QUALIFIED RESPONDENTS
Q203 What is the length of your residency/fellowship program?

[RANGE: 1-5]
[_|_] years

ALL QUALIFIED RESPONDENTS
Q204 How much of your professional time do you spend teaching or training residents/fellows?

1. Almost all of my time
2. Most of my time
3. Some of my time
4. Very little of my time
5. None of my time

ALL QUALIFIED RESPONDENTS
Q205 Does your residency/fellowship program offer supplemental forms of self-directed education in addition to the core curriculum?

1. Yes
2. No

OFFERS SUPPLEMENTAL FORMS OF EDUCATION (Q205r1)
Q205B What type of supplemental forms of self-directed education does your residency/fellowship program offer to residents/fellows?

Please select all that apply.

[MULTI-SELECT, RANDOMIZE]

1. Continuing Medical Education (CME) courses
2. Webinars or web-based modules
3. Conferences
4. Journals
5. Professional association membership/events
6. Other [ANCHOR]

ALL QUALIFIED RESPONDENTS
QC2 For quality control purposes, please select “slightly unhappy” from the list of options below.
1. Very unhappy
2. Slightly unhappy
3. Neutral
4. Slightly happy
5. Very happy

ASSESSMENT OF ADULT/PEDIATRIC GROWTH HORMONE DEFICIENCY (GHD) AND TRAUMATIC BRAIN INJURY (TBI) IN CURRICULA

ALL QUALIFIED RESPONDENTS
Q215 Is teaching residents/fellows about **TBI-induced pituitary disorders** included in your program's curriculum?

1. Yes
2. No

[SHOW Q215B AND Q215C ON SAME SCREEN]

TBI PITUITARY DISORDERS INCLUDED (Q215r1)
Q215B In which types of settings are TBI-induced pituitary disorders taught in your program?

[MULTI-SELECT]

1. During teaching in inpatient rotations
2. Dedicated seminars, lectures, or conferences
3. Precepting in outpatient rotations and continuity clinics
4. Other

TBI PITUITARY DISORDERS INCLUDED (Q215r1)
Q215C Approximately how many hours, on average, are specifically dedicated to TBI-induced pituitary disorders?

[RANGE: 0-500]
1. |_|_| # of hours dedicated to TBI-induced pituitary disorders

ALL QUALIFIED RESPONDENTS
Q225 Is teaching residents/fellows about [INSERT BASED ON PROGRAM "pediatric" if S3Br2 or "adult" if S3Br1] **growth hormone deficiency (GHD) in patients with hypopituitarism <u>following traumatic brain injury (TBI)</u>** included in your program's curriculum?

1. Yes
2. No

[SHOW Q225B AND Q225C ON SAME SCREEN]

GHD FOLLOWING TBI INCLUDED (Q225r1)
Q225B In which types of settings is [INSERT BASED ON PROGRAM "pediatric" if S3Br2 or "adult" if S3Br1] **<u>GHD</u> in patients with hypopituitarism <u>following TBI</u>** covered in your program?

[MULTI-SELECT]

1. During teaching in inpatient rotations
2. Dedicated seminars, lectures, or conference
3. Precepting in outpatient rotations and continuity clinics
4. Other

CONFIDENTIAL

GHD FOLLOWING TBI INCLUDED (Q225r1)
Q225C Approximately how many hours, on average, are specifically dedicated [INSERT BASED ON PROGRAM "pediatric" if S3Br2 or "adult" if S3Br1] **GHD in patients with hypopituitarism following TBI**?

[RANGE: 0-500]

1. |_|_| # of hours dedicated to [INSERT BASED ON PROGRAM "pediatric" if S3Br2 or "adult" if S3Br1] GHD in patients with hypopituitarism following TBI

ALL QUALIFIED RESPONDENTS
Q230 Are any other TBI-induced pituitary disorders covered in your residency/fellowship program?

1. Yes
2. No

[SHOW Q235A AND Q235B ON SAME SCREEN]

ALL QUALIFIED RESPONDENTS
Q235A How important is it to include education/training on hypopituitarism (including GHD) following TBI in your curricula?

1. Very important
2. Fairly important
3. Somewhat important
4. Not at all important

ALL QUALIFIED RESPONDENTS
Q235B How appropriate is it to include education/training on hypopituitarism (including GHD) following TBI in your curricula?

1. Very appropriate
2. Fairly appropriate
3. Somewhat appropriate
4. Not at all appropriate

ALL QUALIFIED RESPONDENTS
Q240 To what extent do you believe [INSERT S3 SPECIALTY TYPE "Endocrinologists", "Neurologists", or "Physiatrists"] should be responsible for…

[COLUMNS]

1. Very responsible
2. Fairly responsible
3. Somewhat responsible
4. Not at all responsible

[ROWS]

1. Screening for hypopituitarism (including GHD) following TBI
2. The early identification and diagnosis of hypopituitarism (including GHD) following TBI
3. The management of hypopituitarism (including GHD) following TBI

4. Patient education about hypopituitarism (including GHD) following TBI
5. Making the decision to refer to other healthcare professionals for management of hypopituitarism (including GHD) following TBI

ALL QUALIFIED RESPONDENTS
Q245 How early do you believe education/training regarding hypopituitarism (including GHD) following TBI should be covered?

Please select one.
1. Within previous education/training prior to residency/fellowship
2. Within the first year of residency (PGY-1)
3. Within the second year of residency (PGY-2)
4. Within the third year of residency or later (PGY-3+)
5. Within fellowship

ALL QUALIFIED RESPONDENTS
Q250 To what extent are each of the following topics related to [INSERT BASED ON PROGRAM "pediatric" if S3Br2 or "adult" if S3Br1] GHD in patients with hypopituitarism following TBI covered during your [INSERT S3 RESPONSE] residency/fellowship program?

[COLUMNS]
1. Great extent
2. Some extent
3. Very little
4. Not at all

[ROWS OF GHD/TBI CORE COMPETENCIES, RANDOMIZE]
1. Screening and diagnosis of GHD following TBI
2. Pathophysiology, symptoms, and clinical characteristics of GHD following TBI
3. Impact of GHD following TBI on daily life
4. Long-term safety and efficacy outcomes of GH substitution
5. The initiation of growth hormone replacement therapy in patients with GHD following TBI
6. The management of growth hormone replacement therapy in patients with GHD following TBI
7. Guidelines for the treatment of GHD following TBI
8. Long-term use of growth hormones and its safety in GHD following TBI
9. Patient education regarding GHD following TBI

GHD COVERED TO SOME EXTENT (Q250c1-3 ANY r1-9)
Q255 For each of the following topics referenced below, indicate the type of education/training that is received.

[COLUMNS, MULTI-SELECT COLUMNS]
1. Inpatient rotations
2. Dedicated seminars, lectures, or conferences
3. Outpatient precepting
4. Other

[SHOW IF SELECTED AT Q250 IN SAME ORDER AS Q250]

1. Screening and diagnosis of GHD following TBI
2. Pathophysiology, symptoms, and clinical characteristics of GHD following TBI
3. Impact of GHD following TBI on daily life
4. Long-term safety and efficacy outcomes of GH substitution
5. The initiation of growth hormone replacement therapy in patients with GHD following TBI
6. The management of growth hormone replacement therapy in patients with GHD following TBI
7. Guidelines for the treatment of GHD following TBI
8. Long-term use of growth hormones and its safety in GHD following TBI
9. Patient education regarding GHD following TBI

ALL QUALIFIED RESPONDENTS
Q257 Based on the education/training provided in your residency/fellowship program, when should screening first occur for GHD in patients after a TBI diagnosis?

1. Within 6 months after TBI
2. Between 6 months and 1 year after TBI
3. More than 1 year after TBI

ALL QUALIFIED RESPONDENTS
Q260 How prepared do you personally feel to do the following?

[COLUMNS]

1. Very prepared
2. Fairly prepared
3. Somewhat prepared
4. Not at all prepared

[ROWS]

1. Provide care/management/treatment for [INSERT BASED ON PROGRAM "pediatric" if S3Br2 or "adult" if S3Br1] GHD in patients with hypopituitarism [SHOW FOR ENDOCRINOLOGY (S100r1)]
2. Provide care/management/treatment for TBI [SHOW FOR NEUROLOGY AND PHYSIATRY (S100r2-3)]
3. Provide care/management/treatment for [INSERT BASED ON PROGRAM "pediatric" if S3Br2 or "adult" if S3Br1] GHD in patients with hypopituitarism following TBI

ALL QUALIFIED RESPONDENTS
Q265 At the end of their residency/fellowship, how prepared do you believe your residents/fellows are to do the following?

[COLUMNS]

1. Very prepared
2. Fairly prepared
3. Somewhat prepared
4. Not at all prepared

[ROWS]

1. Provide care/management/treatment for [INSERT BASED ON PROGRAM "pediatric" if S3Br2 or "adult" if S3Br1] GHD in patients with hypopituitarism [SHOW FOR ENDOCRINOLOGY (S100r1)]
2. Provide care/management/treatment for TBI [SHOW FOR NEUROLOGY AND PHYSIATRY (S100r2-3)]
3. Provide care/management/treatment for [INSERT BASED ON PROGRAM "pediatric" if S3Br2 or "adult" if S3Br1] GHD in patients with hypopituitarism <u>following TBI</u>

ALL QUALIFIED RESPONDENTS
QC3 For quality control purposes, please select "5" from the list of options below.

1. 1
2. 2
3. 3
4. 4
5. 5

ASSESSMENT OF BARRIERS AND OPPORTUNITIES

ALL QUALIFIED RESPONDENTS
Q270 Does your institution have plans to expand formal education of hypopituitarism (including GHD) and its association with TBI in your program?

1. Yes
2. No

HAS PLANS TO EXPAND CURRICULUM (Q270r1)
Q270A Which of the following best describes when you expect to implement or expand your formal education regarding hypopituitarism (including GHD) following TBI?

1. Our program is currently expanding our education/training
2. Within the next year
3. 1-2 years from now
4. More than 2 years from now

ALL QUALIFIED RESPONDENTS
Q275 How much influence do the following professional organizations have regarding the content included within your residency/fellowship curriculum?

[COLUMNS]

1. Great deal of influence
2. Some influence
3. Very little influence
4. No influence at all

[RANDOMIZE, ROWS]

1. American Association of Clinical Endocrinologists (AACE) [SHOW FOR ENDO ONLY (S100r1)]
2. Endocrine Society [SHOW FOR ENDO ONLY (S100r1)]
3. Pituitary Society
4. Growth Hormone Research Society
5. American Neurological Association (ANA) [SHOW FOR NEURO ONLY (S100r2)]
6. American Academy of Neurology (AAN) [SHOW FOR NEURO ONLY (S100r2)]
7. Association of Academic Physiatrists (AAP) [SHOW FOR PHYSIATRY ONLY (S100r3)]
8. American Academy of Physical Medicine and Rehabilitation (AAPM&R) [SHOW FOR PHYSIATRY ONLY (S100r3)]

ALL QUALIFIED RESPONDENTS
Q280 For each the following factors, please indicate to what degree it acts as a barrier to integrating **hypopituitarism (including GHD) following TBI** education into your curriculum.

[COLUMNS]
1. Not a barrier
2. Small barrier
3. Moderate barrier
4. Large barrier

[ROWS, RANDOMIZE]
1. Lack of resident/fellow interest
2. Lack of faculty interest
3. Lack of room (time) in the curriculum
4. Lack of access to trained specialists or faculty expertise
5. Financial limitations
6. Lack of clinical guidelines
7. Little research done on GHD following TBI
8. Inadequate access to research on GHD following TBI
9. Rarity of the condition
10. Not relevant to my medical specialty
11. Other [ANCHOR]

ALL QUALIFIED RESPONDENTS
Q285 How impactful would each of the following resources be in the development of **hypopituitarism (including GHD) following TBI** education in your curriculum?

[COLUMNS]
1. Very impactful
2. Fairly impactful
3. Somewhat impactful
4. Not at all impactful

[ROWS, RANDOMIZE]

1. Clinical guidelines for hypopituitarism (including GHD) following TBI
2. Certification programs for hypopituitarism (including GHD) following TBI
3. Advanced training for hypopituitarism (including GHD) following TBI
4. Resources to expand upon education in residency/fellowship programs
5. Resources for inter-professional education
6. More data on hypopituitarism (including GHD) following TBI
7. Other [ANCHOR AT BOTTOM]
8. For quality control purposes, please select "Somewhat impactful"

ALL QUALIFIED RESPONDENTS
Q290 If there were opportunities for additional learning regarding **hypopituitarism (including GHD) following TBI** outside the standard curriculum, what do you think would be most effective?

Please select all that apply.

[RANDOMIZE, MULTI-SELECT]

1. Continuing Medical Education (CME)
2. Webinars
3. Conferences
4. Journals
5. Professional associations
6. Online resources
7. Other [ANCHOR]

DEMOGRAPHICS

ALL QUALIFIED RESPONDENTS
Q100 Is your residency/fellowship program:

1. Community-Based, Med School Admin
2. Community-Based, Med School Affiliated
3. Community-Based, Non-Affiliated
4. Med School Based
5. Military Program

ALL QUALIFIED RESPONDENTS
Q105 What population/setting does your program consider itself to serve?

Please select all that apply.

[MULTIPLE SELECT]

1. Inner-City
2. Suburban
3. Rural
4. Urban (non-inner city)

ALL QUALIFIED RESPONDENTS
Q110 How many residents/fellows currently participate in your [INSERT S3] residency/fellowship program?

Your best estimate will do.

[RANGE 1-500]
[_|_|_] residents/fellows

ALL QUALIFIED RESPONDENTS
Q150 Thank you for completing this survey! In the next few months, we will be conducting **90-minute virtual focus groups** with individuals like yourself to learn more about the state of education related to endocrine and neurological conditions in residencies/fellowships in the United States.

Are you interested in participating? If so, we will reach back out to you at a later date with more details.

1. Yes, I am interested in participating
2. No, I am not interested in participating
