# Supplementary material for: Coverage of education and training of traumatic brain injury-induced growth hormone deficiency in US residency and fellowship programs: a cross-sectional study
Source: BMC Med Educ. 2024 Jan 10;24:53. doi: 10.1186/s12909-024-05027-8 (PMC10782717; doi:10.1186/s12909-024-05027-8)
Supplement: Supplementary file 2 — Supplementary Material 2: Supplementary Figure 1. Coverage of GHD topics in endocrinology fellowship programs. Supplementary Figure 2. Coverage of GHD topics in neurology residency programs. Supplementary Figure 3. Most effective additional learning opportunities (outside the standard curriculum). Graphical Abstract [file 12909_2024_5027_MOESM2_ESM.pdf]

Additional file 2

Supplementary Figure 1. Coverage of GHD topics in endocrinology fellowship programs

Supplementary Figure 2. Coverage of GHD topics in neurology residency programs

Supplementary Figure 3. Most effective additional learning opportunities (outside the standard curriculum)

Graphical Abstract

Supplementary Figure 1. Coverage of GHD topics in endocrinology fellowship programs

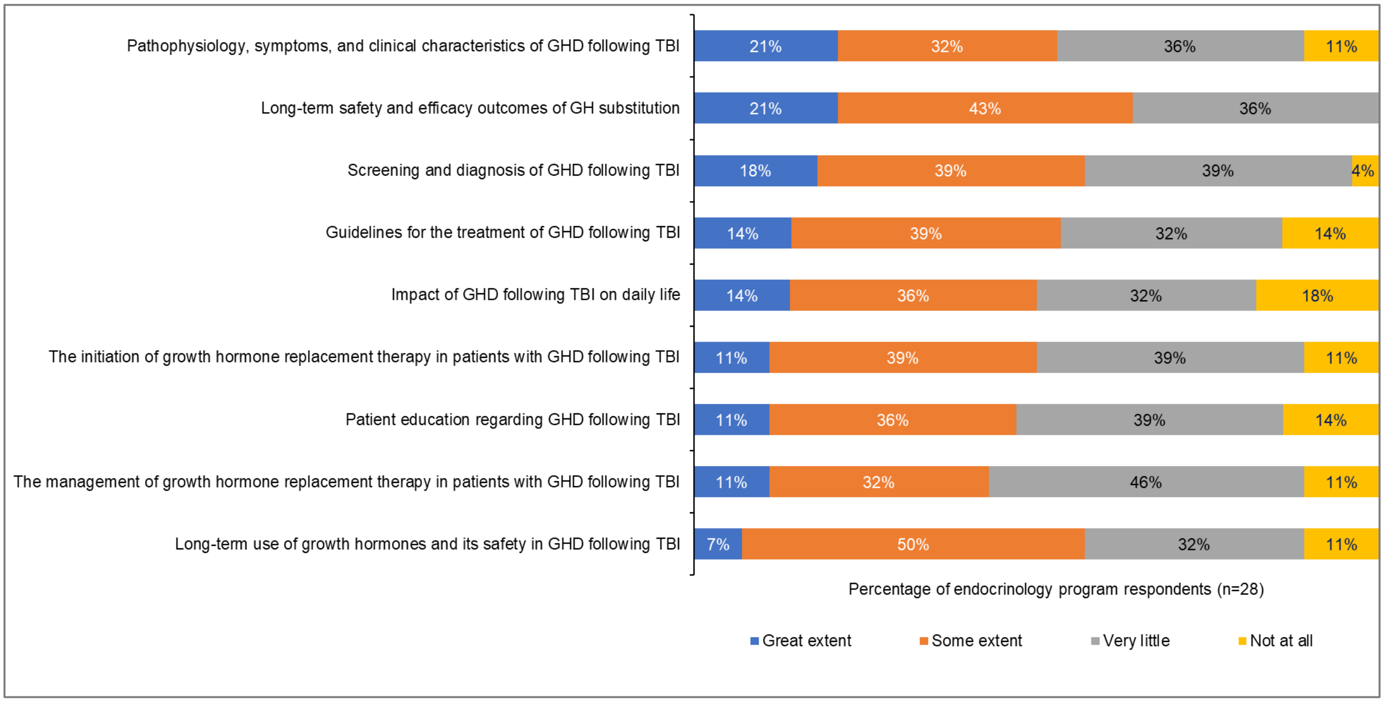

Supplementary Figure 2. Coverage of GHD topics in neurology residency programs

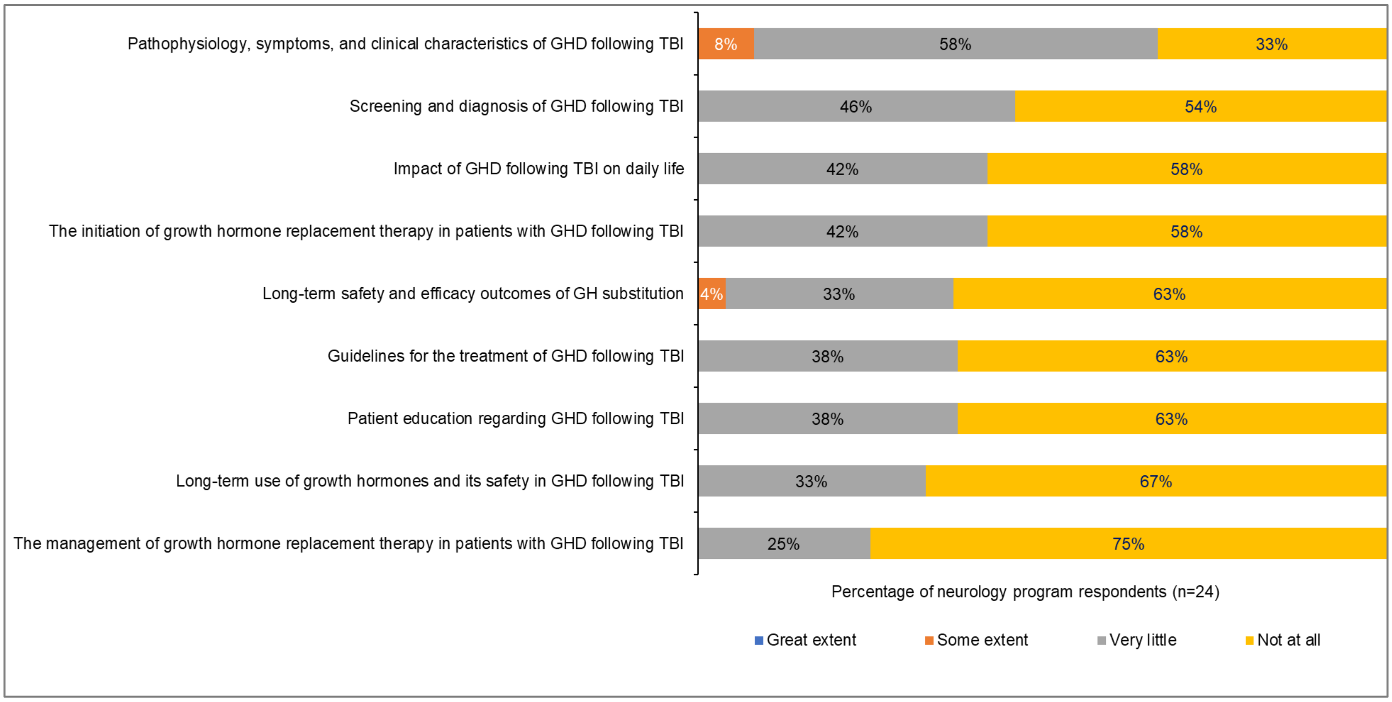

Supplementary Figure 3. Most effective additional learning opportunities (outside the standard curriculum)

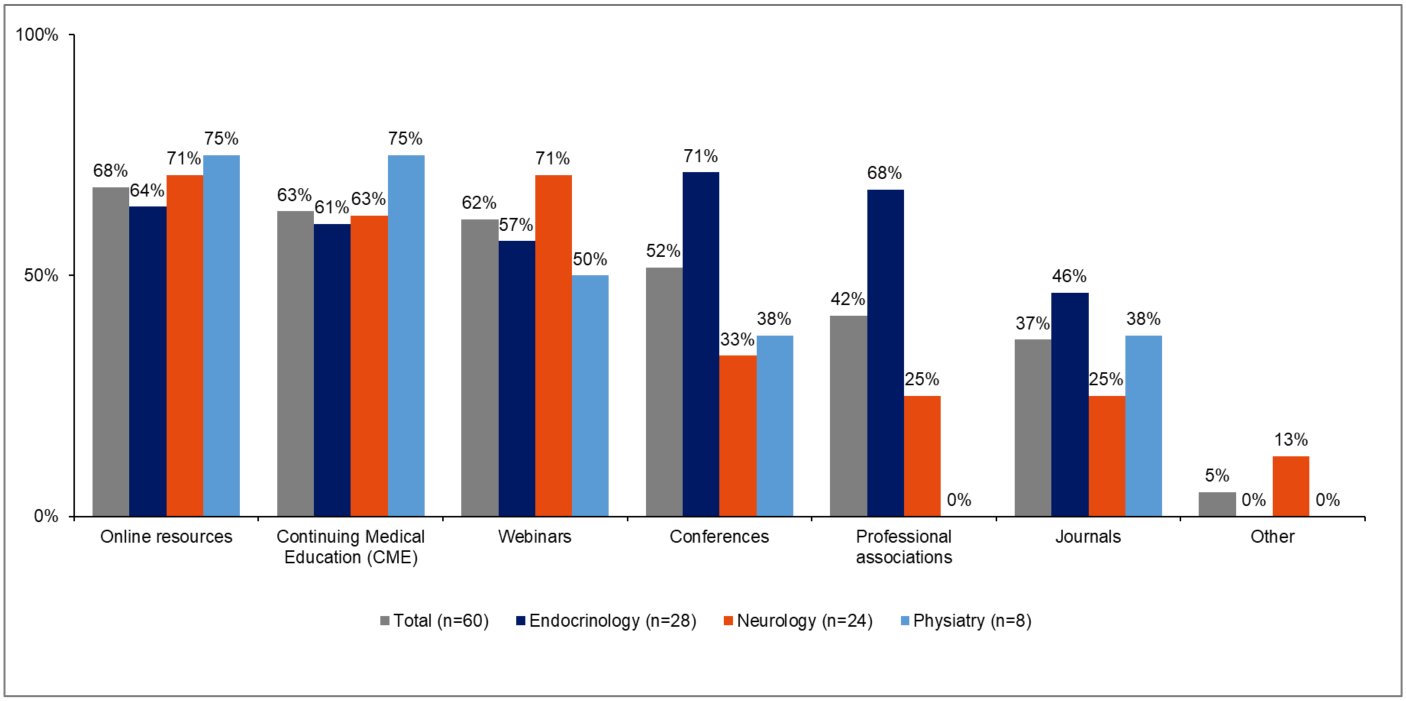

Graphical Abstract

TBI-GHD Curriculum Manuscript Abstract

INTRODUCTION

Growth hormone deficiency (GHD) is a common sequela of traumatic brain injury (TBI). This study explored the education and training opportunities of TBI-induced GHD in US-based postgraduate training program curricula to identify knowledge gaps and opportunities in this area of patient care.

METHODS

Online Survey

Among Postgraduate Directors

endocrinology · neurology · physiatry

Study Field Dates

January 19 - March 28, 2022

RESULTS

419 Fellowship and residency programs were invited to participate

n=60 Program directors completed the survey

ABOUT **50%** Considered TBI-induced hypopituitarism important or fairly important in the curriculum

NEARLY **2/3** Considered TBI-induced hypopituitarism an appropriate training component

IMPORTANCE OF EDUCATION REGARDING HYPOPITUITARISM FOLLOWING TBI

IMPORTANCE: neurology · physiatry · endocrinology

Neurology program directors considered education regarding hypopituitarism following TBI far less important and relevant for their curricula compared with endocrinology and physiatry program directors.

INCLUSION IN CURRICULA

53%

Of the programs responded that they included TBI-induced pituitary disorders in their curricula.

Yet, endocrinology programs included it more frequently than neurology programs.

25% neurology · 68% endocrinology

0% 10% 20% 30% 40% 50% 60% 70% 80%

TOP BARRIERS

- rarity of condition
- lack of room in curriculum

FACTORS IMPACTING DEVELOPMENT OF ADDITIONAL EDUCATIONAL CRITERIA

- consensus clinical guidelines
- availability of more data on the condition

CONCLUSION

To improve the management of TBI-induced GHD, education and training should be expanded in US fellowship programs to prepare trainees to effectively screen, diagnose, and treat TBI-induced GHD.
